# Valorization of Lemon Processing By-Products Through Multi-Strain Fermentation: Strain-Specific Changes in Flavonoids, Limonoids, and Antioxidant Capacity

**DOI:** 10.3390/antiox15060730

**Published:** 2026-06-09

**Authors:** Ching I Lin, Chih Hsuan Fan, Shu Hsien Tsai, Chia Hsin Chang, Chiao Min Yang, Bao Hong Shi, Ying Hsuan Tsai, Ming Yi Lee

**Affiliations:** 1 Department of Nutrition and Health Sciences, Chang Gung University of Science and Technology, Taoyuan 33303, Taiwan; cilin@mail.cgust.edu.tw (C.I.L.);; 2 Research Center for Food and Cosmetic Safety, Chang Gung University of Science and Technology, Taoyuan 33303, Taiwan; 3 Graduate Institute of Health Industry Technology, Chang Gung University of Science and Technology, Taoyuan 33303, Taiwan; 4 Industrial Technology Research Institute, Hsinchu 31057, Taiwan; shuan0331@itri.org.tw (C.H.F.); shtsai@itri.org.tw (S.H.T.);; 5 Center for Drug Research and Development, Chang Gung University of Science and Technology, Taoyuan 33303, Taiwan

**Keywords:** lemon by-products, microbial fermentation, metabolic conversion, strain selection, flavonoids, limonoids, antioxidant activity

## Abstract

Lemon processing by-products are rich in flavonoids, limonoids, and phenolic acids, but their direct utilization is limited by glycoside-dominant flavonoid profiles, bitterness-associated limonoids, and insufficiently defined valorization strategies. This study compared eight food-relevant microorganisms, including lactic acid bacteria, *Bacillus*, yeast, and filamentous fungi, using a common aerobic submerged fermentation framework for lemon by-products. Rather than evaluating fermentation as a single uniform process, the study aimed to determine whether different microbial groups could redirect the same substrate toward distinct functional remodeling profiles. Targeted HPLC analysis of flavonoids, limonoids, and phenolic acids, together with DPPH and ABTS radical-scavenging assays, revealed clear strain-dependent differences in metabolite remodeling and antioxidant outcomes. *L. plantarum* showed the most consistent antioxidant enhancement profile, characterized by increased hesperetin and phenolic acid responses together with low DPPH and ABTS IC_50_ values. *L. pentosus* promoted flavonoid remodeling but showed a more timing-sensitive antioxidant response. *S. cerevisiae* tended to preserve glycosylated flavonoids and showed a release-oriented phenolic acid profile with strong early ABTS activity. *R. stolonifer* exhibited the most pronounced limonoid remodeling, including marked limonin reduction and obacunone accumulation, suggesting potential relevance for bitterness-oriented applications. These findings demonstrate that different microorganisms can be functionally classified according to their dominant remodeling tendencies, including antioxidant enhancement, flavonoid conversion, glycosylated flavonoid preservation, phenolic acid release, and limonoid-associated debittering. This functional classification provides a practical basis for selecting microorganisms according to the intended application of lemon by-product valorization.

## 1. Introduction

*Citrus* fruits are among the major fruit crops worldwide, and lemon is widely processed for juice and related products [1,2]. This processing generates substantial side streams composed mainly of peel, pulp residues, seeds, and pomace, often representing roughly half of the fresh fruit mass [2,3]. Importantly, these materials are not nutritionally exhausted residues; lemon processing by-products remain enriched in flavanones, limonoids, phenolic acids, dietary fiber, and other phytochemicals with technological and functional potential [2,3,4].

However, the valorization of lemon processing by-products remains incomplete. Owing to their high organic load and fermentability, *Citrus* side streams can create economic and environmental burdens when they are discarded or directed only to low-value uses [3,5]. From a circular agriculture perspective, more desirable strategies are those that simultaneously stabilize the biomass and recover or upgrade its value. Among the available options, microbial fermentation is particularly attractive because it is a mild and potentially scalable process that can reshape plant matrices through hydrolysis, release of bound compounds, and generation of new metabolites [5,6]. In this context, fermentation should not be viewed only as a general method for increasing bioactivity, but also as a strain-dependent tool for directing the same by-product substrate toward different functional outcomes, such as antioxidant enhancement, debittering-oriented limonoid remodeling, phenolic acid release, or preservation of selected flavonoid forms [7].

Recent studies have confirmed that fermentation can modify phytochemical profiles and antioxidant properties in *Citrus* matrices, yet the available evidence remains fragmented. Most studies have focused on selected *Lactobacillus* strains in *Citrus* juice, lactic acid bacteria in orange peels, or mixed probiotic consortia in model *Citrus* fermentations [8,9,10]. Even when comparisons are performed, they usually remain within a single microbial class. Therefore, the current knowledge gap is not simply whether citrus by-products can be fermented, but whether taxonomically and functionally different microorganisms drive the same lemon by-product substrate toward distinguishable remodeling trajectories. Previous comparative fermentation studies have shown that different microorganisms can generate different phenolic transformation patterns and antioxidant responses, supporting the need to evaluate microbial category as a determinant of functional output rather than treating fermentation as a single uniform process [11]. Direct side-by-side evaluation of taxonomically distinct food-relevant microorganisms under a unified fermentation framework therefore remains limited, particularly for lemon processing by-products.

For lemon processing by-products, three groups of metabolites are especially relevant. First, *Citrus* flavanones are typically dominated by glycosidic forms such as hesperidin and narirutin, and glycosylation can constrain absorption relative to the corresponding aglycone forms [12,13]. Microbial or enzymatic deglycosylation of citrus flavonoid glycosides has been reported to increase aglycone forms such as hesperetin and naringenin, indicating that flavonoid remodeling can be functionally meaningful beyond changes in total flavonoid content alone [14]. Second, limonoids are important contributors to *Citrus* bitterness and therefore strongly influence palatability and application potential [15,16]. Third, phenolic acids provide informative markers of microbial remodeling because fermentative microorganisms can hydrolyze, decarboxylate, reduce, or otherwise transform food phenolics in a strain-dependent manner [17,18].

Based on these functional considerations, hesperidin, hesperetin, and narirutin were selected as representative flavonoids, limonin, nomilin, and obacunone as bitterness-related limonoids, and *p*-coumaric, ferulic, and vanillic acids as indicators of phenolic acid remodeling. Simultaneous tracking of these compounds provided a focused way to evaluate fermentation-induced changes related to flavonoid conversion, limonoid-associated bitterness, phenolic acid remodeling, and antioxidant performance. These targeted compounds were used as functional markers rather than as a complete chemical mass balance of the lemon by-product substrate.

The eight selected microorganisms represented major food-relevant fermentation groups, including lactic acid bacteria, *Bacillus*, yeast, and filamentous fungi. These groups were included because they may differ in their abilities to release, preserve, transform, or deplete phytochemicals during fermentation; yeast fermentation of lemon matrices has also been reported to alter phenolic compounds, organic acids, antioxidant activity, and sensory-related characteristics [19]. Therefore, the present study was not designed to identify a single universally superior microorganism, but to determine whether different microbial groups could direct the same lemon by-product substrate toward distinct functional profiles. Using a common substrate-based fermentation framework, temporal high-performance liquid chromatography (HPLC)-based metabolite profiling and DPPH/ABTS antioxidant assays were applied to support strain selection according to intended valorization goals, such as antioxidant enhancement, limonoid-associated debittering, phenolic acid release, or preservation of glycosylated flavonoids.

## 2. Materials and Methods

### 2.1. Raw Material and Preparation of Lemon By-Product Substrate

Lemon processing by-products (cultivar: *Eureka*) were obtained from the Yongda Food Technology Co., Ltd. (Neipu Township, Pingtung County, Taiwan) in February 2024. The by-products consisted of peel, pulp residues, and seeds remaining after juice extraction. Upon receipt, the material was immediately stored at −20 °C to minimize compositional changes prior to pretreatment. A single batch of lemon processing by-products was used throughout the experiment to minimize batch-to-batch variation in substrate composition. For experimental use, the by-products were dried in a hot-air oven at 70 °C to constant weight (DO-60, DENGYNG Instruments Co., Ltd., New Taipei City, Taiwan), milled using a milling device (RT-02A, Rong Tsong Precision Technology Co., Taichung, Taiwan), and passed through a 0.25 mm sieve to obtain a homogeneous powder. The powder was sealed and stored at 4 °C until use.

For submerged fermentation, lemon by-product powder was suspended in deionized water at a solid-to-liquid ratio of 1:20 (*m*/*v*), sterilized at 121 °C for 15 min, cooled to room temperature, and used as the common fermentation matrix for all strains. Using the same sterilized substrate matrix was intended to minimize substrate-related variation and to allow strain-associated fermentation profiles to be compared under a shared substrate and analytical framework.

### 2.2. Microorganisms and Pre-Culture Conditions

#### 2.2.1. Strains and Source

Eight food-relevant microorganisms were obtained from the Bioresource Collection and Research Center (BCRC, Hsinchu City, Taiwan): *Lactobacillus paracasei* BCRC 17484, *Lactobacillus plantarum* BCRC 10357, *Lactobacillus pentosus* BCRC 12944, *Lactobacillus bulgaricus* BCRC 10696, *Leuconostoc mesenteroides* BCRC 12580, *Bacillus subtilis* BCRC 11602, *Saccharomyces cerevisiae* BCRC 20579, and *Rhizopus stolonifer* BCRC 31135. These strains were selected to represent major food-relevant microbial groups, including lactic acid bacteria, *Bacillus*, yeast, and filamentous fungi, which may differ in their capacities for metabolite release, hydrolysis, conversion, and matrix remodeling. Strain names are reported according to the BCRC designations, and abbreviated names are used thereafter.

#### 2.2.2. Pre-Culture and Stock Preservation

Each strain was revived and propagated under the incubation conditions listed in Table 1. The pre-culture media and temperatures followed the recommended growth conditions for each strain and were used to obtain active inocula rather than to compare growth performance across microbial groups. For bacteria and yeast, activated cultures were mixed 1:1 (*v*/*v*) with sterile 50% glycerol to prepare cryostocks [20]. For *R. stolonifer*, an approximately 1 cm mycelial plug was aseptically transferred into a sterile screw-cap tube containing 25% glycerol in phosphate-buffered saline (PBS) and stored at −20 °C [21]. The media and incubation conditions used for inoculum preparation are summarized in Table 1.

#### 2.2.3. Growth Assessment in Routine Culture Media

To characterize strain-specific growth profiles and determine suitable harvest times for inoculum preparation, each activated strain was inoculated into 9 mL of its corresponding culture medium (Table 1). Aliquots were collected at 0, 24, 48, 72, and 96 h. After serial dilution, viable counts were determined by the spread plate method using plates containing 25–250 colonies and were expressed as colony-forming units (CFU)/mL. Because the filamentous morphology of *R. stolonifer* precluded reliable enumeration by plate counting, this strain was not included in CFU-based comparisons [8]. These growth measurements were used only to guide inoculum preparation and were not used as an inferential comparison of microbial fitness among strains, because each strain was propagated in its own routine culture medium.

### 2.3. Unified Aerobic Submerged Fermentation of Lemon By-Products

To enable direct comparison among microbial groups, all strains were fermented in the same sterilized lemon by-product substrate described in Section 2.1. Pre-cultures were harvested during the logarithmic phase and used as inocula. The harvest time for each inoculum was selected according to the growth assessment described in Section 2.2.3 and the strain-specific growth conditions listed in Table 1. For bacteria and yeast, inoculum densities were adjusted to approximately 1 × 10^8^ CFU/mL based on serial dilution and plate counting, and 4 mL of inoculum was added to 100 mL of sterile substrate in 250 mL Erlenmeyer flasks, corresponding to an inoculation ratio of 4% (*v*/*v*). For *R. stolonifer*, an actively growing pre-culture was used as inoculum because reliable CFU standardization was not feasible for this filamentous strain; 100 µL of actively growing pre-culture was added per 100 mL of sterile substrate. An uninoculated sterile substrate incubated in parallel under the same conditions served as the unfermented lemon by-product control (UFLP). The UFLPs received an equal volume of the corresponding sterile medium instead of microbial inoculum and were incubated under the same strain-specific temperature conditions as the fermented samples. Therefore, the corresponding temperature-matched UFLP was used as the baseline reference for time-dependent changes in the sterilized lemon by-product matrix at each sampling day.

Fermentations were conducted under aerobic conditions at the strain-specific temperatures listed in Table 1. Fermentations were carried out in 250 mL Erlenmeyer flasks containing 100 mL of substrate under aerobic shaking conditions at 100 rpm using a constant-temperature incubator (CIB-70, KF, Taichung City, Taiwan; Cat. No. S2701-CIB). Strain-specific incubation temperatures were used to maintain active growth and metabolism for each microorganism; therefore, the results were interpreted as functional fermentation profiles under each strain’s applicable growth condition rather than as a temperature-independent ranking. The common elements of the fermentation design included the same lemon by-product substrate, sterilization procedure, solid-to-liquid ratio, inoculum ratio for bacteria and yeast, aerobic submerged format, sampling period, extraction procedure, and analytical workflow.

A day 0 sterilized substrate sample was collected before incubation to represent the initial targeted metabolite profile, and subsequent samples were collected from day 1 to day 7. At each sampling point, fermentation broths were concentrated under reduced pressure at 60 °C to less than 50 mL using a rotary evaporator (N-1300, EYELA Co., Tokyo, Japan), frozen at −30 °C, and lyophilized to constant weight. The dried materials were then ground, sealed, and stored at 4 °C until extraction and analysis. All fermentations were performed using three independent biological replicates. Each biological replicate represented an independently inoculated fermentation flask.

### 2.4. Preparation of Ethanol Extracts

Lyophilized fermented samples and the corresponding unfermented controls were extracted with 75% aqueous ethanol at a solid-to-solvent ratio of 1:20 (*m*/*v*). Each mixture was sonicated in an ultrasonic bath (KQ-500DE, Kunshan Ultrasonic Instruments Co., Kunshan, China; frequency 40 kHz, power 500 W) for 30 min and centrifuged at 8600 rpm and 4 °C for 10 min. The residue was re-extracted once under the same conditions. Supernatants were combined, vacuum-filtered, concentrated under reduced pressure, and lyophilized. The dried extracts were stored at 4 °C until antioxidant assays and targeted compound analysis. The same extraction procedure was applied to all fermented samples and UFLPs to ensure that antioxidant activity and targeted metabolite contents were compared on the basis of similarly prepared ethanol extracts.

### 2.5. Targeted Quantification of Flavonoids, Limonoids, and Phenolic Acids

Targeted compound analysis was performed by the Research Center for Food and Cosmetic Safety, Chang Gung University of Science and Technology, using ultra-performance liquid chromatography–electrospray ionization tandem mass spectrometry (UPLC-ESI-MS/MS). For sample preparation, 1 mg of each dried ethanol extract was reconstituted in 2 mL of methanol and filtered through a 0.22 μm polyvinylidene fluoride (PVDF) membrane prior to analysis.

The target analytes comprised three flavonoids (hesperidin, hesperetin, and narirutin), three limonoids (nomilin, limonin, and obacunone), and three phenolic acids (*p*-coumaric acid, ferulic acid, and vanillic acid). These analytes were selected because they are directly related to the major functional questions of this study, namely flavonoid remodeling, limonoid-associated bitterness, phenolic acid conversion, and antioxidant performance. Sugars and organic acids were not quantified in the present targeted method and were therefore not included in the chemical profile analysis.

Chromatographic analysis was performed using a Waters Acquity UPLC system coupled with a TQS triple quadrupole mass spectrometer (Waters, Milford, MA, USA). Separation was achieved on a bridged ethylene hybrid (BEH) C18 column (100 mm × 2.1 mm, 1.7 μm) maintained at 25 °C with a flow rate of 0.3 mL/min. The mobile phase composition, gradient elution program, and mass spectrometric parameters were adopted from the validated method reported [22].

Compound identification and quantification were carried out according to the reference method using authentic standards and multiple reaction monitoring (MRM) analysis. Identification was based on comparison with authentic standards using retention time and MRM transitions, and quantification was performed using standard-based calibration according to the referenced method. Authentic standards of hesperidin, hesperetin, narirutin, nomilin, limonin, obacunone, *p*-coumaric acid, ferulic acid, and vanillic acid were purchased from Sigma-Aldrich (St. Louis, MO, USA) and had HPLC purity of ≥98%. Unless otherwise stated, compound contents are reported as ng/mg dry extract. Baseline targeted metabolite profiles were obtained from the UFLP samples analyzed using the same extraction and UPLC-ESI-MS/MS procedures. Compounds below the quantifiable range were reported as not detected (ND) and were interpreted cautiously as being below the detection or quantification capability of the targeted method.

### 2.6. DPPH Radical-Scavenging Assay

DPPH radical-scavenging activity was determined with minor modifications of a previously described method [23]. Briefly, dried extracts were dissolved in 75% ethanol and serially diluted over a concentration range suitable for IC_50_ determination. The concentration of each test solution was calculated on the basis of dry ethanol extract weight. Then, 140 μL of sample solution was mixed with 560 μL of 0.5 mM 2,2-diphenyl-1-picrylhydrazyl (DPPH) solution and incubated in the dark for 30 min at room temperature. Absorbance was measured at 517 nm in 96-well microplates using a microplate reader (PowerWave XS2, BioTek Instruments, Winooski, VT, USA), with 75% ethanol as the blank. Sample blanks containing the same extract concentration without DPPH were measured and subtracted to correct for sample background absorbance.

Radical-scavenging activity was calculated as follows:

(1) $$\mathrm{Scavenging}\;\mathrm{activity}\;(\%)\;=\;\frac{{Abs}_{blank,\;517\;}-{\;Abs}_{sample,\;517}}{{Abs}_{blank,\;517}}\times 100$$

Antioxidant capacity was expressed as the concentration required to achieve 50% inhibition (IC_50_, mg/mL), with lower IC_50_ values indicating stronger radical-scavenging activity. The reported IC_50_ values therefore represent mg dry ethanol extract/mL required to reach 50% radical-scavenging activity and do not represent the concentration of any individual flavonoid, limonoid, or phenolic acid. IC_50_ values were calculated by linear interpolation between the two concentrations immediately above and below 50% scavenging activity.

### 2.7. ABTS Radical-Scavenging Assay

ABTS radical-scavenging activity was determined with minor modifications of a previously described method [24]. 2,2′-azino-bis(3-ethylbenzothiazoline-6-sulfonic acid) (ABTS) (96 mg) was dissolved in 20 mL of water and mixed with 5 mL of 2.45 mM potassium persulfate. The mixture was kept in the dark at room temperature for 12–16 h to generate the ABTS radical cation (ABTS•+). Before use, the ABTS•+ solution was diluted with water to an absorbance of 0.70 ± 0.02 at 734 nm.

For analysis, 10 μL of appropriately diluted sample in 75% ethanol was mixed with 1 mL of ABTS•+ solution, incubated in the dark at room temperature for 6 min, and measured at 734 nm. The concentration of each test solution was calculated on the basis of dry ethanol extract weight. Seventy-five percent ethanol served as the blank. Radical-scavenging activity was calculated as follows:

(2) $$\mathrm{Scavenging}\;\mathrm{activity}\;(\%)\;=\;\frac{{Abs}_{blank,\;734}\;-\;{Abs}_{sample,\;734}}{{Abs}_{blank,\;734}}\times 100$$

Antioxidant capacity was expressed as IC_50_ (mg/mL), with lower IC_50_ values indicating stronger radical-scavenging activity. As in the DPPH assay, ABTS IC_50_ values are reported as mg dry ethanol extract/mL and reflect the radical-scavenging activity of crude extracts rather than individual quantified compounds. IC_50_ values were calculated by linear interpolation between the two concentrations immediately above and below 50% scavenging activity.

### 2.8. Statistical Analysis

All data are presented as mean ± standard deviation (SD) from three independent biological replicates. For fermentation experiments, each biological replicate corresponded to an independently inoculated fermentation flask. For the fermentation experiment, the effects of microbial strain, fermentation time, and their interaction on metabolite contents and antioxidant activity were evaluated by two-way analysis of variance (ANOVA). When significant effects were detected, multiple comparisons were performed using Tukey’s post hoc test for within-strain temporal comparisons and adjusted multiple-comparison testing against the corresponding UFLP at the same sampling day. Because lower IC_50_ values indicate stronger antioxidant activity, decreases in DPPH or ABTS IC_50_ were interpreted as increased radical-scavenging capacity.

Because each strain was cultured in its own routine medium during pre-culture, viable count data were used descriptively to characterize growth profiles and were not subjected to inferential comparison across strains. Pearson correlation analysis was used as an exploratory approach to evaluate linear associations between targeted metabolites and antioxidant indices across all samples. Correlation results were used to support integrated interpretation of metabolite–function associations and were not interpreted as direct evidence of causal biochemical pathways. All statistical analyses were performed using GraphPad Prism 9 (GraphPad Software, San Diego, CA, USA), and statistical significance was set at *p* < 0.05.

## 3. Results and Discussion

### 3.1. Growth Profiles in Routine Culture Media

All strains proliferated after inoculation, but their growth patterns differed in their respective routine culture media (Table 2). *Lactobacillus paracasei* and *Lactobacillus plantarum* showed rapid early growth, reaching 1.54 × 10^9^ and 2.30 × 10^9^ CFU/mL, respectively, at 24 h. Thereafter, *L. plantarum* maintained cell counts in the 10^8^ CFU/mL range through 96 h, whereas *L. paracasei* declined markedly by 96 h. *L. pentosus*, *Leuconostoc mesenteroides*, and *L. bulgaricus* also reached high counts within 24 h and then progressively declined, consistent with entry into stationary and subsequent death phases. Among the non-lactic acid microorganisms, *Bacillus subtilis* reached a lower maximum cell density (3.60 × 10^7^ CFU/mL at 24 h) and then remained relatively stable, whereas *Saccharomyces cerevisiae* showed delayed proliferation and reached its highest count at 72 h (6.10 × 10^9^ CFU/mL). Because *Rhizopus stolonifer* is filamentous, it was not included in CFU-based comparisons. These measurements were obtained in strain-specific routine media and were used primarily to guide inoculum preparation rather than to infer growth performance in the lemon by-product substrate. Therefore, the growth data in Table 2 were used only to guide inoculum preparation and should not be interpreted as a direct comparison of strain competitiveness in the lemon by-product fermentation matrix.

### 3.2. Effects of Fermentation on Compound Profiles in Lemon By-Products

The targeted HPLC profiles showed that fermentation did not generate a single uniform response across microorganisms. Instead, the same lemon by-product substrate was redirected toward different metabolite-remodeling trajectories depending on the microbial group. Therefore, the results are interpreted below according to functional remodeling patterns—flavonoid conversion or preservation, limonoid-targeted remodeling, phenolic acid release or depletion, and antioxidant-related outcomes—rather than as a simple ranking of individual strains.

#### 3.2.1. Flavonoids

**Figure 1:**
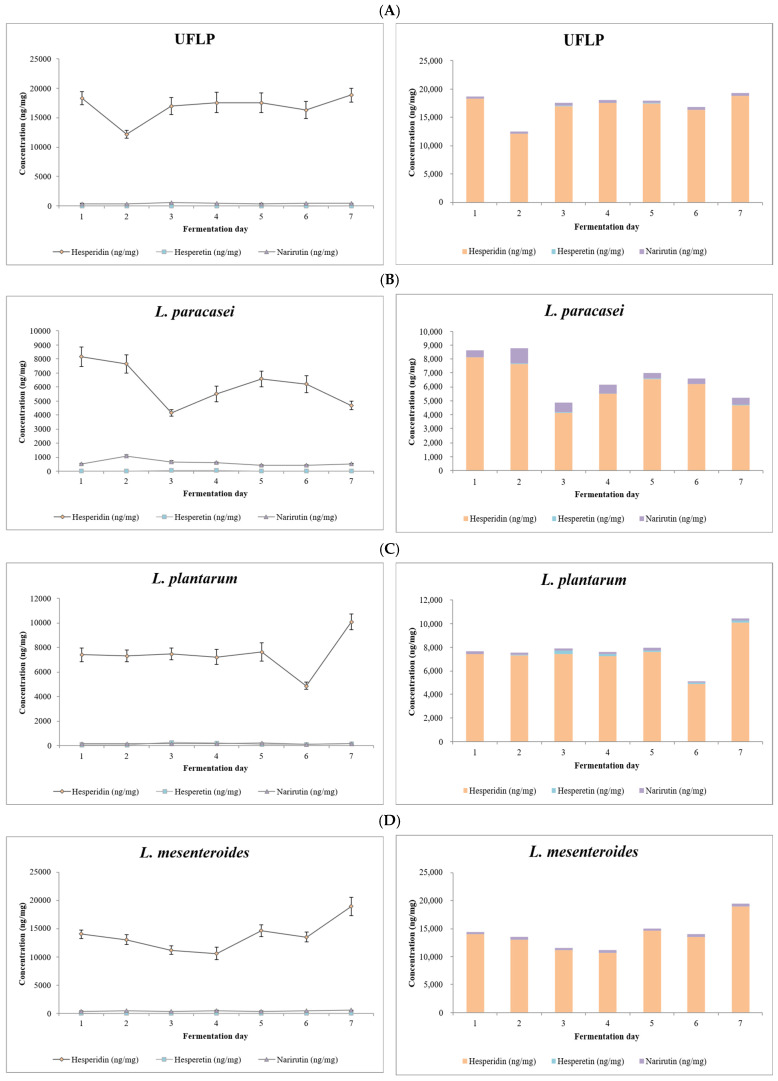

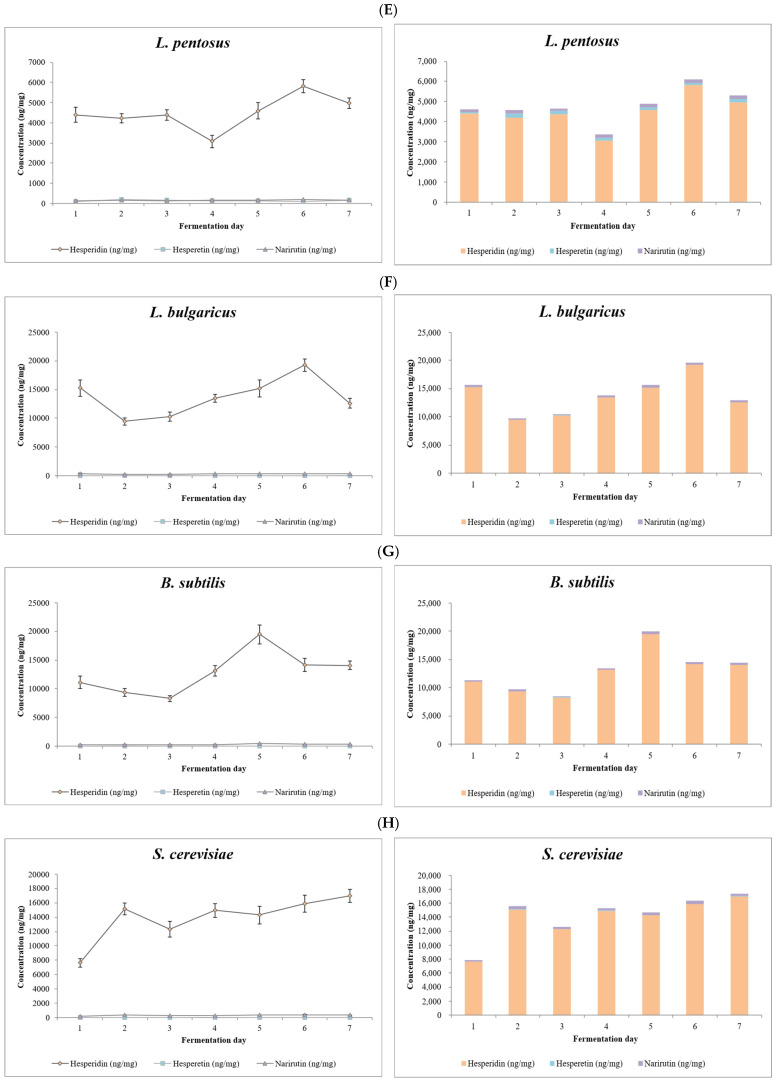

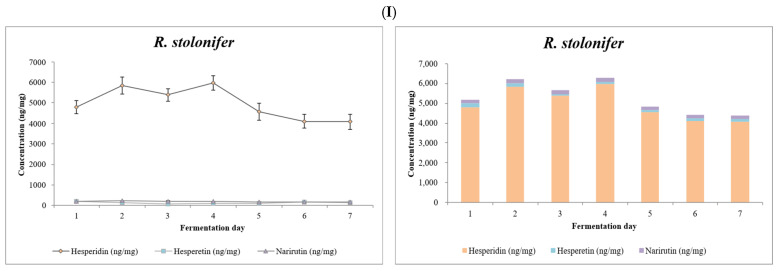
Changes in flavonoid composition during fermentation of lemon by-products by different microbial strains. (**A**) Unfermented lemon by-product control (UFLP); (**B**) *Lactobacillus paracasei* (*L. paracasei*); (**C**) *Lactobacillus plantarum* (*L. plantarum*); (**D**) *Leuconostoc mesenteroides* (*L. mesenteroides*); (**E**) *Lactobacillus pentosus* (*L. pentosus*); (**F**) *Lactobacillus bulgaricus* (*L. bulgaricus*); (**G**) *Bacillus subtilis* (*B. subtilis*); (**H**) *Saccharomyces cerevisiae* (*S. cerevisiae*); and (**I**) *Rhizopus stolonifer* (*R. stolonifer*). The left panels show temporal changes in hesperidin, hesperetin, and narirutin during the 7-day fermentation period, and the right panels show the corresponding cumulative contents as stacked bar charts. Data are presented as mean ± standard deviation (SD) (*n* = 3). UFLP = unfermented lemon by-product control.

Flavonoid composition changed during fermentation and showed clear strain-dependent remodeling patterns (Figure 1, Table S1). In the unfermented lemon by-product control (UFLP), hesperidin remained the dominant flavonoid throughout the 7-day period, with only minor fluctuations, indicating relative stability under sterile conditions. This point is important because fermentation can change flavonoid profiles through both matrix-level release of bound compounds and enzymatic transformation of glycosylated forms, rather than simply increasing or decreasing total flavonoid content [25].

Among the tested strains, lactic acid bacteria—particularly *L. plantarum* and *L. pentosus*—showed the strongest flavonoid conversion patterns, characterized by reduced hesperidin and increased hesperetin levels. *L. plantarum* exhibited the most pronounced increase in hesperetin, reaching 279.82 ± 25.16 ng/mg on day 3, while *L. pentosus* showed a similar but earlier increase. These results indicate that the lactic acid bacterial group, especially *L. plantarum* and *L. pentosus*, tended to redirect lemon by-product flavonoids toward an aglycone-enriched profile. From a biochemical perspective, this type of conversion is plausible because citrus flavanone glycosides such as hesperidin contain rhamnosyl and glucosyl moieties, and their complete conversion to hesperetin generally requires cleavage of these sugar residues by α-L-rhamnosidase and/or β-D-glucosidase activities [26]. This interpretation is consistent with previous studies showing that *L. plantarum*-associated processing can convert citrus flavonoid glycosides such as hesperidin and naringin into the corresponding aglycones hesperetin and naringenin [14], and that *L. pentosus* strains may possess α-L-rhamnosidase and β-D-glucosidase activities involved in flavonoid biotransformation [27]. The β-glucosidase activity of *L. plantarum* has also been reviewed as an important functional trait in food fermentation, supporting its possible role in releasing or transforming plant-derived glycosides [28]. However, because glycosidase activity was not directly measured in the present study, the observed increase in hesperetin should be interpreted as metabolite-level evidence consistent with deglycosylation rather than direct enzymatic proof.

In contrast, *S. cerevisiae* maintained relatively high hesperidin levels throughout fermentation, particularly on day 7 (17,000 ± 905.30 ng/mg), indicating limited depletion of the dominant glycosylated flavonoid and a comparatively preservation-oriented profile. This yeast-associated pattern suggests that *S. cerevisiae* may be more suitable when preservation of extractable glycosylated flavonoids is desired, rather than when extensive conversion to aglycones is the primary goal. This interpretation is supported by studies on *S. cerevisiae*-fermented orange systems, in which hesperidin remained a major phenolic compound and antioxidant capacity was largely preserved, while fermentation altered the broader metabolite and bioaccessibility profiles [29]. A similar application-oriented distinction has been reported in fermentation studies showing that different microorganisms can generate different phenolic transformation and antioxidant profiles from the same phenolic substrates [11].

Meanwhile, *R. stolonifer* showed elevated hesperetin levels mainly during the early fermentation stage, suggesting rapid early-stage flavonoid release or transformation. This response was distinct from the more sustained lactic-acid-bacterial conversion pattern and may represent an early-release type of flavonoid remodeling. This interpretation is consistent with reports that filamentous-fungal fermentation can enhance flavonoid accumulation in citrus peel matrices and that fermentation time is a key determinant of flavonoid accumulation and antioxidant activity [30]. A recent orange-peel study using *R. stolonifer* also reported increases in total flavonoid content and broad metabolite remodeling after fungal fermentation, supporting the classification of *R. stolonifer* as a matrix-remodeling fungus rather than only a hydrolytic converter [31]. Because fungal enzyme activities were not assayed, this finding should be described as an early extractable-metabolite response associated with fungal fermentation rather than confirmed enzymatic hydrolysis.

Compared with hesperidin and hesperetin, narirutin showed relatively minor changes across most strains, indicating that fermentation effects were both strain- and compound-dependent rather than uniform across all citrus flavonoids. Overall, the flavonoid results revealed three distinct metabolic tendencies: aglycone-oriented conversion by *L. plantarum* and *L. pentosus*, preservation of glycosylated flavonoids by *S. cerevisiae*, and early-stage flavonoid release or remodeling by *R. stolonifer*. This distinction is application-relevant because aglycone-enriched profiles may be preferred for bioactivity-oriented extracts, whereas preservation of glycosylated flavonoids may be useful when maintaining the original citrus flavonoid profile or extractable flavonoid reservoir is desirable [13,14,26]. Thus, strain selection can be used to guide the flavonoid profile of fermented lemon by-products according to the intended product function.

#### 3.2.2. Limonoids

**Figure 2:**
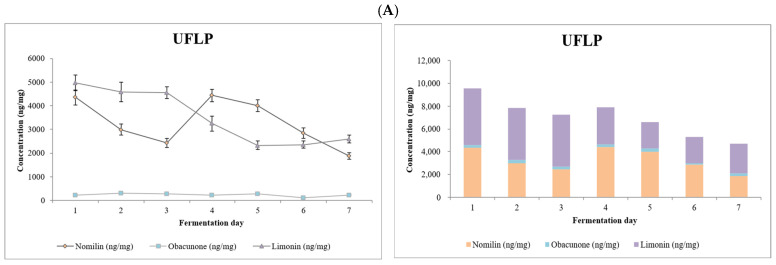

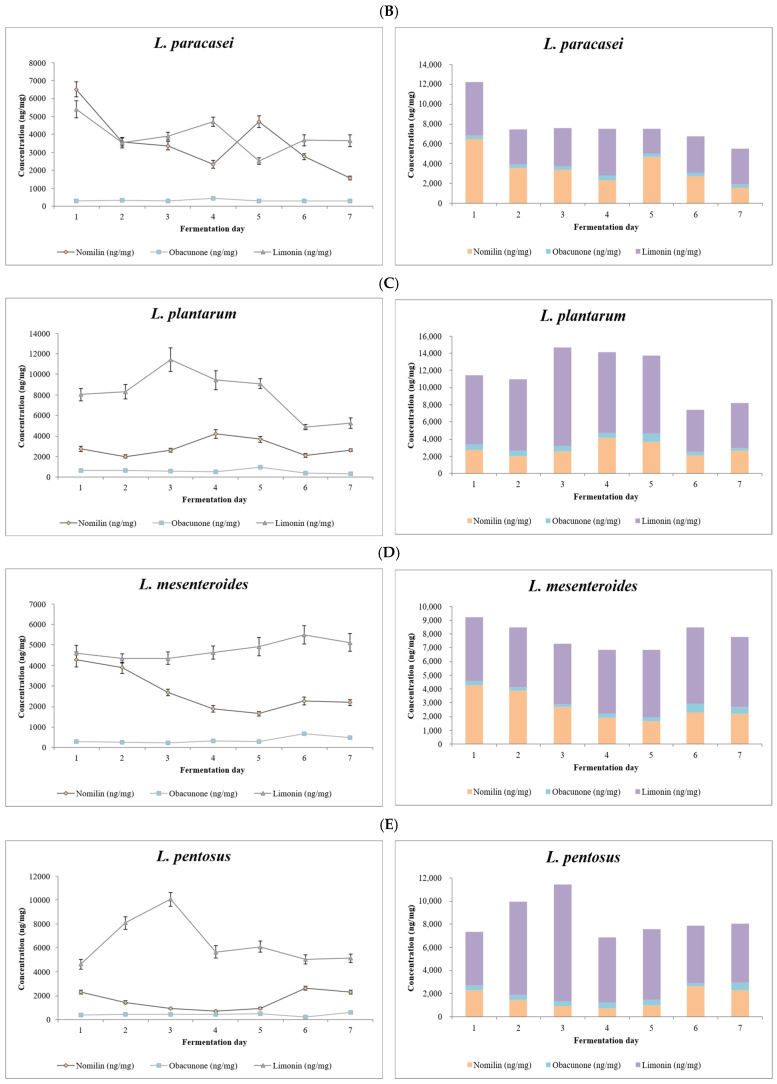

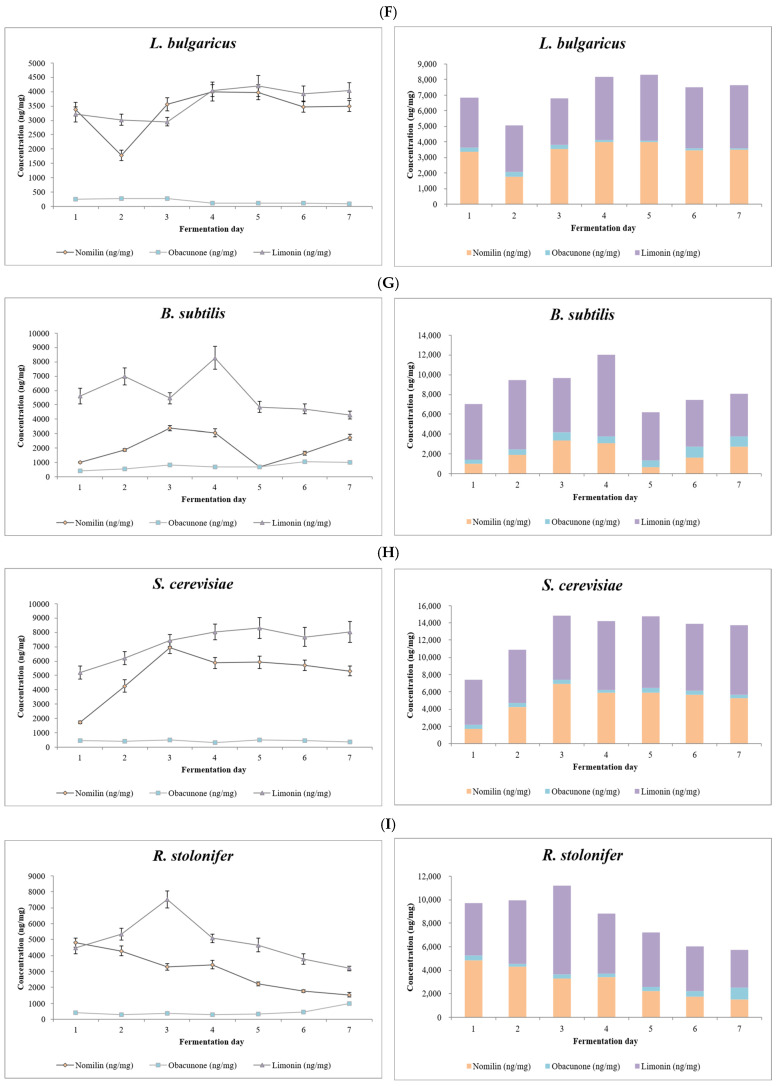
Changes in limonoid composition during fermentation of lemon by-products by different microbial strains. (**A**) Unfermented lemon by-product control (UFLP); (**B**) *Lactobacillus paracasei* (*L. paracasei*); (**C**) *Lactobacillus plantarum* (*L. plantarum*); (**D**) *Leuconostoc mesenteroides* (*L. mesenteroides*); (**E**) *Lactobacillus pentosus* (*L. pentosus*); (**F**) *Lactobacillus bulgaricus* (*L. bulgaricus*); (**G**) *Bacillus subtilis* (*B. subtilis*); (**H**) *Saccharomyces cerevisiae* (*S. cerevisiae*); and (**I**) *Rhizopus stolonifer* (*R. stolonifer*). The left panels show temporal changes in obacunone, nomilin, and limonin during the 7-day fermentation period, and the right panels show the corresponding cumulative contents as stacked bar charts. Data are presented as mean ± standard deviation (SD) (*n* = 3). UFLP = unfermented lemon by-product control.

The three limonoids—limonin, nomilin, and obacunone—also showed strain-dependent changes during fermentation (Figure 2. In UFLP, the limonoid profile remained relatively stable throughout the 7-day period, indicating limited non-microbial alteration under sterile conditions. In contrast, fermentation generated distinct limonoid remodeling patterns, ranging from transient conversion-like changes and compound depletion to extensive limonin reduction. These results indicate that limonoid remodeling represented a separate functional axis from flavonoid conversion and antioxidant enhancement. This distinction is important because citrus limonoids are highly oxygenated triterpenoids whose structural forms are closely related not only to bitterness perception but also to the value and utilization potential of citrus-derived ingredients [32].

Among the tested strains, *L. plantarum* and *L. pentosus* showed the most evident limonoid conversion patterns. Both strains exhibited transient increases in limonin accompanied by changes in nomilin and obacunone. *L. pentosus* also exhibited a limonin peak on days 2–3, then stabilized at moderate levels, with obacunone steadily increasing and nomilin rebounding later. These patterns suggest active limonoid remodeling rather than simple accumulation or disappearance. Similar microbial limonoid transformations have been reported in bacterial systems, including conversion of nomilin to obacunone and limonin-related metabolism, indicating that changes among limonoid aglycones can occur through microbial biotransformation [33,34]. However, because limonoid intermediates, enzyme activities, and complete mass balance were not determined, these changes should be described as conversion-oriented metabolite profiles rather than definitive biochemical pathways.

In contrast, *L. paracasei* showed lower limonin and nomilin levels than the control at several time points (*p* < 0.05), indicating net depletion of major limonoids during fermentation. This pattern differed substantially from the transient conversion behavior observed in *L. plantarum* and *L. pentosus*. The observed depletion may reflect microbial metabolism, reduced extractability, adsorption to microbial biomass, or conversion into non-target limonoid derivatives; these possibilities cannot be distinguished using the present targeted HPLC data alone. Such caution is necessary because previous citrus debittering studies have shown that limonin reduction may occur through different routes, including microbial degradation, enzymatic conversion, immobilized-cell systems, or adsorption-based removal, and these mechanisms cannot be differentiated without pathway-specific assays [34,35,36].

*B. subtilis* showed transient limonin accumulation mid fermentation with rising obacunone, indicating moderate conversion, whereas *L. mesenteroides*, *L. bulgaricus*, and *S. cerevisiae* exhibited comparatively modest changes in limonoid composition, suggesting limited involvement in limonoid remodeling under the conditions tested. This minor-response group further supports the application-oriented interpretation of the study: food-relevant microorganisms should not be assumed to have equivalent limonoid-remodeling capacity simply because they are able to grow or ferment plant substrates.

Among all strains, *R. stolonifer* was most distinctive: limonin dropped to its lowest level by day 7 (3188.25 ± 162.46 ng/mg, *p* < 0.05) as obacunone rose to 978.93 ± 76.52 ng/mg. Compared with the other microorganisms, *R. stolonifer* demonstrated the strongest limonin reduction and the most pronounced shift in limonoid composition. This profile suggests that *R. stolonifer* may be useful as a limonoid-remodeling strain when the intended application is to reduce bitter limonoid-related components rather than to maximize antioxidant activity. The practical relevance of this profile is supported by citrus-processing studies showing that limonin can be reduced by microbial or enzymatic systems, including limonoate dehydrogenase-producing bacteria and immobilized-cell approaches [35,37,38]. This interpretation is consistent with the broader use of microbial or enzymatic processing for citrus debittering, including naringinase-based strategies and limonin-related processing approaches [36,39].

Because limonin is recognized as one of the major contributors to citrus bitterness, the marked reduction observed in *R. stolonifer* may have practical implications for debittering of lemon by-products. Sensory studies have shown that limonin and nomilin are closely associated with bitterness perception in citrus juice systems. In orange juice, reported recognition thresholds were 4.7 mg/L for limonin and 2.6 mg/L for nomilin, and the two compounds may interact in bitterness perception depending on the matrix composition [26,36]. Nevertheless, the present limonoid data are expressed as ng/mg dry extract, and therefore cannot be directly converted to sensory bitterness thresholds without information on product formulation, dilution, matrix composition, and final serving concentration. Thus, the decrease in limonin should be interpreted as chemical evidence of potential debittering relevance, not as confirmed sensory debittering.

Overall, microbial fermentation yielded clear limonoid metabolic divergence: *L. plantarum* and *L. pentosus* mainly promoted conversion-oriented profiles, *L. paracasei* showed net depletion of major limonoids, and *R. stolonifer* showed the strongest limonin-reduction profile. These findings support the central concept that different microbial groups can be selected for different lemon by-product applications: *R. stolonifer* may be prioritized for limonoid-targeted remodeling, whereas other strains may be more suitable for antioxidant or flavonoid-oriented outcomes. In this context, *R. stolonifer* should be positioned as a debittering-oriented candidate, whereas *L. plantarum* and *L. pentosus* should be interpreted as broader conversion-oriented strains. This distinction supports a practical strain-selection strategy rather than a single-strain ranking approach.

#### 3.2.3. Phenolic Acids

**Figure 3:**
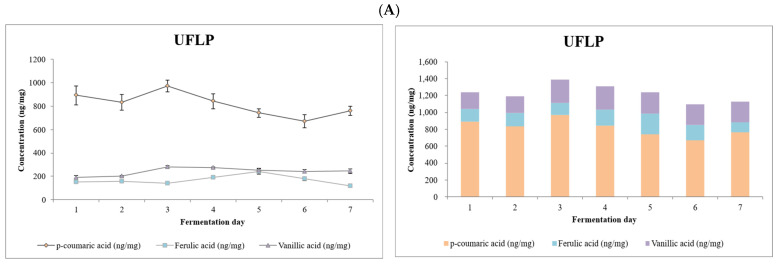

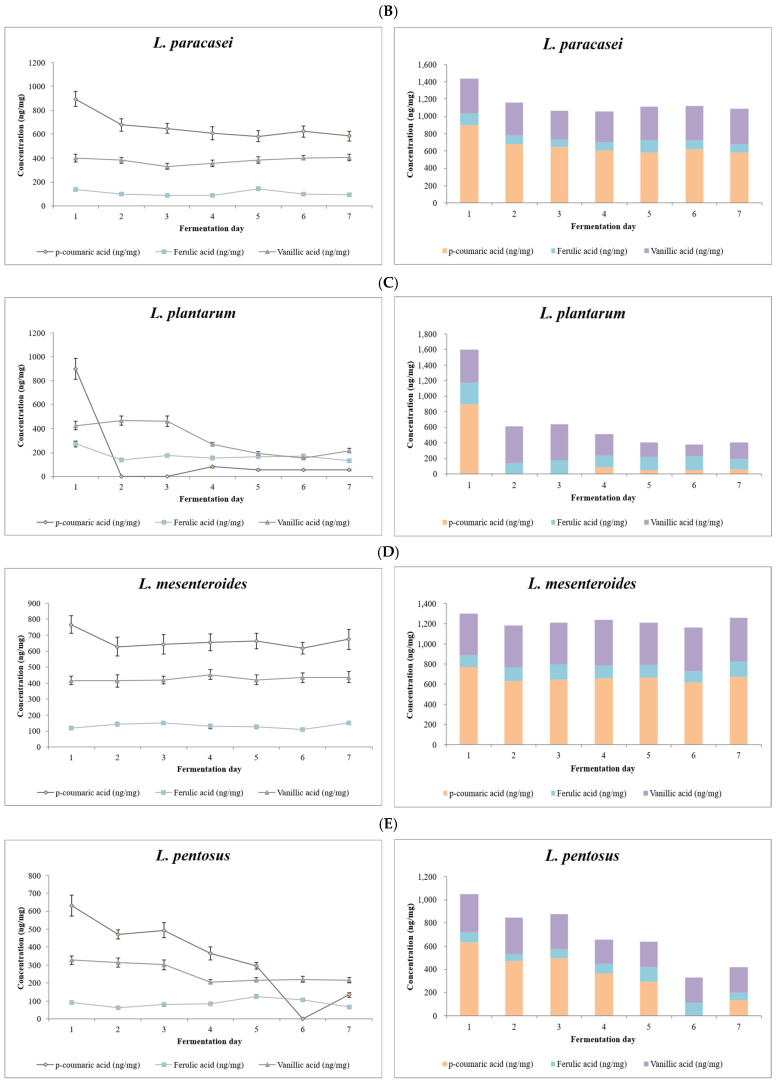

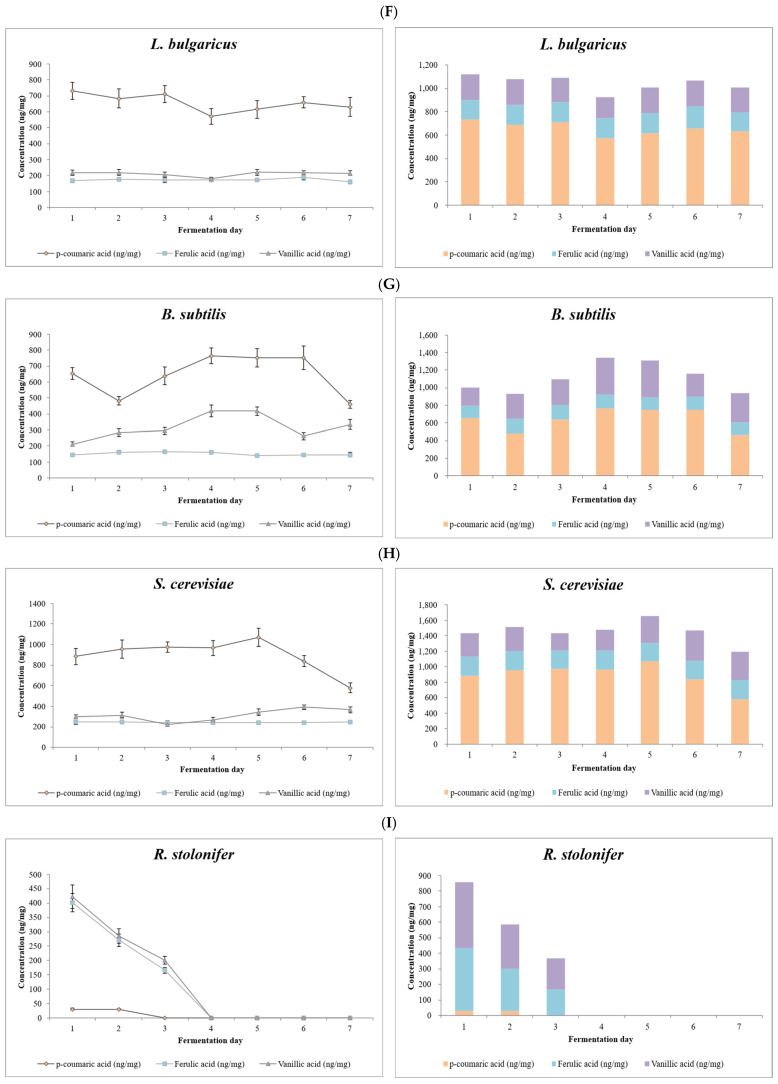
Changes in phenolic acid composition during fermentation of lemon by-products by different microbial strains. (**A**) Unfermented lemon by-product control (UFLP); (**B**) *Lactobacillus paracasei* (*L. paracasei*); (**C**) *Lactobacillus plantarum* (*L. plantarum*); (**D**) *Leuconostoc mesenteroides* (*L. mesenteroides*); (**E**) *Lactobacillus pentosus* (*L. pentosus*); (**F**) *Lactobacillus bulgaricus* (*L. bulgaricus*); (**G**) *Bacillus subtilis* (*B. subtilis*); (**H**) *Saccharomyces cerevisiae* (*S. cerevisiae*); and (**I**) *Rhizopus stolonifer* (*R. stolonifer*). The left panels show temporal changes in *p*-coumaric acid, ferulic acid, and vanillic acid during the 7-day fermentation period, and the right panels show the corresponding cumulative contents as stacked bar charts. Data are presented as mean ± standard deviation (SD) (*n* = 3). UFLP = unfermented lemon by-product control.

Phenolic acids also exhibited strong strain-dependent changes (Figure 3). In UFLP, concentrations of *p*-coumaric, ferulic, and vanillic acids did not vary significantly (*p* > 0.05), indicating stability under sterile conditions aside from spontaneous reactions. In contrast, fermentation generated distinct phenolic acid remodeling patterns, ranging from active remodeling and metabolite accumulation to rapid depletion. Together with the flavonoid and limonoid results, these data show that microbial fermentation redirected lemon by-products into different chemical profiles rather than producing a uniform increase in all bioactive compounds. This distinction is important because phenolic acids in plant matrices may occur as free, esterified, glycosylated, or cell-wall-bound forms, and fermentation can alter their extractability, release, and conversion in a strain- and substrate-dependent manner [40].

Among the tested microorganisms, *L. plantarum* and *L. pentosus* showed the clearest phenolic acid remodeling patterns. *L. plantarum* showed higher *p*-coumaric acid early, followed by a rapid decline to low levels, with transient accumulation of ferulic acid and a brief rise in vanillic acid. *L. pentosus* showed the clearest *p*-coumaric acid depletion pattern, with *p*-coumaric acid falling to not detected (ND) by day 6, while ferulic and vanillic acids remained detectable. Rather than indicating a simple linear conversion from *p*-coumaric acid to ferulic or vanillic acid, these patterns suggest dynamic release, utilization, and transformation of hydroxycinnamic and hydroxybenzoic acid derivatives during fermentation. This interpretation is more appropriate than a strict upstream–downstream pathway because hydroxycinnamic acids such as *p*-coumaric and ferulic acids can undergo decarboxylation, reduction, ester hydrolysis, or conversion into non-target derivatives depending on the microorganism and fermentation matrix [40,41]. This interpretation is consistent with studies showing that *L. plantarum* can metabolize food phenolics through enzyme systems involving esterases, decarboxylases, and reductases [17], and that hydroxycinnamic acid metabolism in lactobacilli commonly involves decarboxylation and reduction reactions [18]. More specifically, *p*-coumaric acid decarboxylase from *L. plantarum* has been biochemically characterized and shown to decarboxylate hydroxycinnamic acids including *p*-coumaric, caffeic, and ferulic acids, supporting the plausibility of hydroxycinnamic acid remodeling in the present lactic acid bacteria (LAB)-fermented samples [42]. Because these enzymes were not measured in the present study, the proposed mechanism remains a literature-supported interpretation rather than direct pathway confirmation.

In contrast, *L. paracasei* showed immediate decreases in *p*-coumaric acid from day 1 (−35% vs. UFLP, *p* < 0.05). This pattern differed from the conversion-oriented profiles observed in *L. plantarum* and *L. pentosus* and may indicate preferential utilization of upstream phenolic acids during fermentation. Alternatively, the decrease may reflect reduced extractability or conversion into non-target phenolic derivatives that were not included in the present UPLC-MS/MS panel. This cautious interpretation is important because targeted analysis of only three phenolic acids cannot distinguish between microbial consumption, conversion into volatile phenols or reduced derivatives, binding to the residual matrix, or formation of other non-target metabolites [40].

Among the non-lactic acid microorganisms, *S. cerevisiae* showed the highest *p*-coumaric acid levels (day 5 > 1000 ng/mg), with stable ferulic acid and moderate vanillic acid, suggesting a net release- or accumulation-oriented phenolic acid profile rather than rapid depletion under the present conditions. This release-oriented profile agrees with previous reports showing that yeast fermentation of lemon juice can increase phenolic compounds and antioxidant activity, although the specific response depends on yeast strain and substrate matrix [19]. This interpretation is also consistent with studies on *S. cerevisiae*–fermented orange systems showing that yeast fermentation can alter phenolic profiles and phenolic bioaccessibility, while maintaining substantial levels of citrus phenolics [29]. At the same time, *S. cerevisiae* should not be regarded only as a passive releasing organism, because *PAD1* and *FDC1* have been shown to be essential for the decarboxylation of phenylacrylic acids, including *p*-coumaric and ferulic acids, in *S. cerevisiae* [42].

By comparison, *R. stolonifer* showed low phenolic acid levels that quickly disappeared during fermentation. This pattern may reflect fungal utilization, oxidative transformation, reduced extractability, or conversion into non-target metabolites rather than simple disappearance of phenolic compounds. This interpretation is consistent with studies showing that *Rhizopus*-based fermentation can substantially reshape phenolic acid profiles in agro-industrial matrices, with changes depending on substrate composition, enzyme activity, and fermentation time [43,44]. Importantly, this phenolic acid depletion occurred alongside strong limonoid remodeling, suggesting a functional trade-off: *R. stolonifer* may be useful for limonoid-targeted remodeling but less suitable for preserving phenolic acid-associated antioxidant capacity during prolonged fermentation. Therefore, the value of *R. stolonifer* fermentation may depend strongly on process timing: a shorter fermentation may preserve early radical-scavenging activity, whereas prolonged fermentation may favor limonoid remodeling at the expense of phenolic acid retention.

Meanwhile, *L. mesenteroides*, *L. bulgaricus* and *B. subtilis* showed limited fluctuations across all three phenolic acids, with only minor *p*-coumaric decreases at a few time points, implying weaker involvement in this pathway. Although *B. subtilis* displayed a transient increase in vanillic acid during days 4–5, this response was not sufficient to define a clear phenolic acid remodeling type under the present fermentation conditions. This minor-response group further supports the strain-selection concept of the study, because food-relevant microorganisms did not show equivalent capacities to remodel phenolic acids even when applied to the same lemon by-product substrate.

Collectively, microbial fermentation generated distinct phenolic acid remodeling strategies. *L. plantarum* and *L. pentosus* showed active LAB-associated remodeling, particularly *p*-coumaric acid depletion with detectable ferulic and vanillic acid responses, whereas *S. cerevisiae* favored net release or maintenance and *R. stolonifer* showed rapid phenolic acid depletion. Therefore, phenolic acid data further support the application-oriented classification of strains: antioxidant-oriented fermentation may favor strains that retain or generate phenolic acids, whereas limonoid-remodeling strains may not necessarily preserve antioxidant-related phenolics. This application-oriented interpretation is supported by the general antioxidant relevance of phenolic and polyphenolic compounds in food matrices, although antioxidant performance of crude extracts ultimately depends on the combined contribution of multiple compounds rather than any single phenolic acid [45]. Because only three phenolic acids were targeted, the present results should be interpreted as focused indicators of phenolic remodeling rather than a complete phenolic mass balance.

### 3.3. Effects of Fermentation on Antioxidant Capacity of Lemon By-Products

Across strains, DPPH IC_50_ values ranged from 1.30 ± 0.07 to 4.40 ± 0.35 mg/mL, and ABTS IC_50_ values ranged from 11.96 ± 0.69 to 42.22 ± 2.84 mg/mL, indicating significant effects of both strain and fermentation time on antioxidant performance (ANOVA, *p* < 0.05) (Table 3). Because DPPH and ABTS differ in radical chemistry, solvent compatibility, reaction kinetics, and sensitivity toward different antioxidant structures, the two assays should be interpreted as complementary but not interchangeable chemical indices of radical-scavenging capacity [23,46,47]. Therefore, DPPH and ABTS were used to compare the radical-scavenging behavior of crude ethanol extracts, but the results should not be interpreted as direct evidence of biological antioxidant efficacy in food or physiological systems [46].

In UFLP, DPPH scavenging was initially strong on day 1 (1.34 ± 0.08 mg/mL) but deteriorated by day 7 (3.60 ± 0.31 mg/mL, *p* < 0.05), suggesting time-dependent loss, transformation, or reduced extractable contribution of radical-scavenging components under non-fermented conditions. ABTS IC_50_ values remained relatively stable between 21 and 26 mg/mL, suggesting that antioxidant capacity under non-fermented conditions depended mainly on compound stability rather than active microbial remodeling.

Among all tested strains, *L. plantarum* showed the most consistent and superior antioxidant activity: DPPH IC_50_ dropped to a minimum on day 5 (1.30 ± 0.07 mg/mL) and remained low through day 7; ABTS IC_50_ stabilized at 14–18 mg/mL. This superior antioxidant activity corresponded with the accumulation of hesperetin and phenolic acids observed in previous sections. Thus, *L. plantarum* can be classified as the antioxidant-enhancement type among the tested microorganisms. This classification is consistent with citrus fermentation studies showing that *Lactobacillus* fermentation can increase total phenolics, total flavonoids, and antioxidant activity in citrus matrices, although the direction and magnitude of the response depend on the strain and fermentation time [8,9]. Mechanistically, fermentation may enhance antioxidant activity by releasing bound phenolics, transforming glycosides into more active aglycone forms, or generating new antioxidant-related metabolites [7,14]. However, in the present study, this mechanism is inferred from targeted metabolite trends and literature rather than directly confirmed by enzyme assays or activity-guided fractionation.

In contrast, *L. pentosus* and *L. bulgaricus* showed weaker performance: the former’s DPPH IC_50_ rose significantly in mid-fermentation (4.40 ± 0.35 mg/mL), and the latter peaked on day 6 (4.23 ± 0.37 mg/mL), with parallel increases in ABTS IC_50_. These results suggest that the conversion or depletion of antioxidant compounds may have exceeded the generation of new antioxidant metabolites during fermentation. This finding is important because it shows that flavonoid conversion alone did not guarantee improved antioxidant performance; antioxidant outcomes depended on the balance among flavonoids, phenolic acids, limonoids, and fermentation duration. This result is consistent with the broader principle that antioxidant activity of crude plant extracts is determined by the combined effects of multiple compounds, their structures, concentrations, degradation products, and possible synergistic or antagonistic interactions, rather than by the increase in one metabolite class alone [45,46].

Among non-lactic acid microbes, *S. cerevisiae* showed modest changes in DPPH IC_50_ (2.00–3.12 mg/mL) but the lowest ABTS IC_50_ on day 1 (11.96 ± 0.69 mg/mL), followed by higher values during days 4–6 and partial recovery by day 7. This pattern suggests an early ABTS-responsive profile, possibly associated with the release or maintenance of water-soluble antioxidant constituents. A similar yeast-related response has been reported in fermented lemon and orange matrices, where yeast fermentation altered phenolic compounds, organic acids, antioxidant activity, and phenolic bioaccessibility, but the specific response depended on the yeast strain and substrate matrix [19,29]. Together with its preservation of hesperidin and release-oriented phenolic acid pattern, *S. cerevisiae* may be considered a preservation/release-oriented strain rather than a strong aglycone conversion strain. However, because ABTS is a chemical assay and the extract contained multiple compound classes, the early ABTS response should not be assigned to a single metabolite without further fractionation or activity-guided analysis.

*R. stolonifer* demonstrated good early DPPH scavenging (~1.5 mg/mL) but worsened later (3.57 ± 0.28 mg/mL, *p* < 0.05); ABTS IC_50_ peaked on day 4 (42.22 ± 2.84 mg/mL), the weakest performance among all strains. This decline coincided with the rapid depletion of targeted phenolic acids and strong limonoid remodeling observed in previous sections. These results suggest a clear functional trade-off: *R. stolonifer* showed the strongest potential for limonoid remodeling and limonin reduction, but this was not accompanied by preservation of antioxidant capacity during prolonged fermentation.

Overall, *L. plantarum* delivered the most stable and robust antioxidant activity across both assays. *S. cerevisiae* showed an early ABTS-responsive profile, while *L. pentosus*, *L. bulgaricus*, and *R. stolonifer* showed weaker or time-sensitive antioxidant responses. These findings reinforce the main message of this study: fermentation of lemon by-products should not be interpreted as a universal antioxidant enhancement process. Instead, each microorganism generated a distinct functional trajectory, and the appropriate strain should be selected according to the intended application. This application-oriented interpretation is particularly important for by-product valorization, because the preferred fermentation endpoint may differ depending on whether the target is antioxidant enriched extracts, preservation of citrus flavonoids, phenolic acid release, or limonoid-associated debittering.

### 3.4. Functional Classification of Microbial Remodeling Profiles and Metabolite-Antioxidant Associations

#### 3.4.1. Functional Classification of Microbial Remodeling Profiles

Synthesizing the compound profiles with antioxidant outcomes shows clear, strain-specific metabolic differentiation and functional divergence during fermentation of lemon by-products (Table 4). Rather than producing uniform responses, different microorganisms favored distinct metabolic trajectories associated with flavonoid remodeling, limonoid transformation, phenolic acid metabolism, and antioxidant enhancement. Accordingly, the tested strains can be grouped according to their dominant application-oriented functions rather than ranked by a single overall performance index. The detailed day-by-day variation trends and statistical directions for individual compounds and antioxidant indices are provided in Supplementary Table S1.

*L. plantarum* represented the antioxidant enhancement type. It combined strong hesperetin accumulation, active phenolic acid remodeling, and consistently low DPPH and ABTS IC_50_ values after the early fermentation stage. Therefore, *L. plantarum* appears most suitable when the intended goal is to produce antioxidant-oriented fermented lemon by-product extracts.

*L. pentosus* represented a flavonoid-conversion but timing-sensitive type. Although it promoted strong flavonoid remodeling and *p*-coumaric acid depletion, its mid-fermentation antioxidant performance was weaker than that of *L. plantarum*. This indicates that conversion of selected metabolites does not necessarily translate into stronger antioxidant activity unless fermentation time is carefully controlled.

*S. cerevisiae* represented a preservation/release-oriented type. It maintained a relatively high level of hesperidin and showed phenolic acid release or accumulation, together with a strong early ABTS response. This profile suggests potential value when preservation of glycosylated flavonoids or early-stage release of soluble antioxidant-related compounds is preferred.

*R. stolonifer* represented the limonoid-remodeling type. It showed the most pronounced limonin reduction and obacunone increase, indicating potential relevance for bitterness-related applications. However, this profile was accompanied by rapid phenolic acid depletion and late-stage antioxidant loss, suggesting that *R. stolonifer* should be considered a debittering-oriented candidate rather than a general antioxidant enhancement strain.

The remaining strains, including *L. paracasei*, *L. mesenteroides*, *L. bulgaricus*, and *B. subtilis*, showed moderate, limited, or less favorable remodeling patterns under the present conditions. Their results are still useful because they indicate that not all food-relevant microorganisms provide the same value in lemon by-product fermentation.

Taken together, the integrated results support a functional selection framework: *L. plantarum* may be selected for antioxidant enhancement, *R. stolonifer* for limonoid-targeted remodeling, *S. cerevisiae* for preservation/release-oriented profiles, and *L. pentosus* for flavonoid conversion when fermentation timing is controlled.

#### 3.4.2. Pearson Correlation Analysis

**Figure 4:**
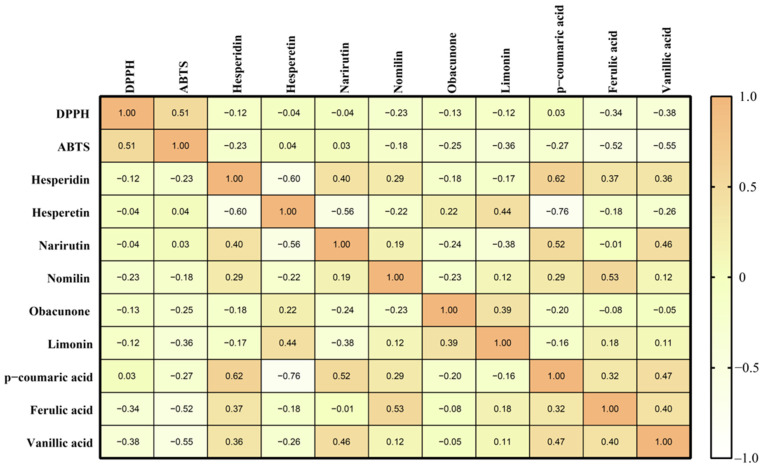
Exploratory Pearson correlation analysis among targeted metabolites and antioxidant indices in fermented lemon by-product samples. Darker colors indicate higher absolute correlation coefficients (r), with black and white corresponding to positive and negative correlations, respectively. Correlation analysis was used to evaluate metabolite–function associations and was not interpreted as direct evidence of causal biochemical pathways.

Within flavonoids (Figure 4), hesperidin correlated negatively with its aglycone hesperetin (r = −0.602, *p* < 0.001), and narirutin correlated negatively with hesperetin (r = −0.557, *p* < 0.001), confirming the reciprocal relationship between declining glycosides and rising aglycones. This association supports the interpretation of flavonoid remodeling across the strain-time dataset, especially in *L. plantarum*, *L. pentosus*, and *R. stolonifer*. However, because the correlation analysis pooled samples across strains and fermentation days, these associations should be interpreted as overall co-variation rather than direct evidence of a single biochemical pathway within each microorganism. This interpretation follows the general statistical principle that correlation analysis can identify the strength and direction of association between variables, but it cannot by itself establish causal or mechanistic relationships.

Hesperidin also correlated positively with *p*-coumaric acid (r = 0.623, *p* < 0.001), suggesting synchronous release during fermentation. This relationship may reflect shared matrix release, co-extraction behavior, or strain-dependent preservation of extractable phenolic constituents, but it does not establish direct metabolic conversion between the two compounds [25,40].

Across limonoids and other compounds, hesperetin correlated positively with limonin (r = 0.444, *p* < 0.001), and nomilin correlated positively with ferulic acid (r = 0.531, *p* < 0.001), suggesting possible associations between limonoid remodeling and phenolic acid changes. These associations are useful for identifying coordinated remodeling patterns, but they should be used for hypothesis generation rather than pathway confirmation because limonoid intermediates and enzyme activities were not measured. Therefore, the positive correlations involving limonoids should be interpreted as coordinated strain–time remodeling patterns, not as evidence that hesperetin, limonin, nomilin, or ferulic acid are directly connected through the same metabolic pathway.

Within the phenolic acid set, ferulic acid correlated positively with vanillic acid (r = 0.398, *p* < 0.01), and *p*-coumaric acid correlated positively with vanillic acid (r = 0.469, *p* < 0.001), suggesting coordinated phenolic acid remodeling during fermentation rather than a confirmed sequential conversion pathway. These positive correlations may reflect simultaneous release from the plant matrix, shared preservation under specific fermentation conditions, or partial downstream transformation; targeted pathway experiments would be required to distinguish among these possibilities.

For antioxidant indices, DPPH IC_50_ correlated negatively with ferulic acid (r = −0.336, *p* < 0.01) and vanillic acid (r = −0.375, *p* < 0.01), and ABTS IC_50_ correlated negatively with ferulic acid (r = −0.520, *p* < 0.001) and vanillic acid (r = −0.549, *p* < 0.001). Because lower IC_50_ values indicate stronger radical-scavenging activity, these negative correlations indicate that higher ferulic and vanillic acid levels were associated with stronger antioxidant capacity in the crude extracts [45]. DPPH and ABTS were also positively correlated (r = 0.512, *p* < 0.001), indicating consistent evaluation trends between the two assays. Nevertheless, the moderate correlation also indicates that the two assays captured overlapping but not identical antioxidant responses, reinforcing the need to interpret them as complementary rather than interchangeable assays.

Taken together, the observed correlations support the proposed relationships among flavonoid deglycosylation, limonoid transformation, and phenolic acid remodeling during fermentation. The correlation results strengthen the functional classification proposed in Table 4, but they do not replace enzyme assays, untargeted metabolomics, or mass balance analysis. Therefore, the main contribution of this study is the comparative mapping of strain-specific remodeling profiles and antioxidant performance, which can guide future strain selection for lemon by-product valorization. In this context, Pearson analysis provides supporting evidence for metabolite–function associations, while Table 4 translates these associations into an application-oriented strain selection framework.

### 3.5. Study Limitations and Application Considerations

Several limitations should be considered when interpreting these results. First, the present study used targeted HPLC analysis of selected flavonoids, limonoids, and phenolic acids, but did not include untargeted metabolomics, carbohydrate profiling, organic acid profiling, or complete mass balance analysis. Therefore, the observed changes should be interpreted as targeted remodeling signatures rather than complete biochemical transformation pathways. Second, enzyme activities were not directly measured; mechanistic explanations involving glycosidases, esterases, decarboxylases, reductases, or other microbial enzymes are therefore literature-supported interpretations rather than direct experimental confirmation. Third, antioxidant activity was evaluated using DPPH and ABTS chemical assays only. These assays are useful for comparing radical-scavenging capacity of crude extracts, but they do not fully represent biological antioxidant effects in food or physiological systems. Fourth, although *R. stolonifer* markedly reduced limonin, sensory evaluation was not performed, and the limonoid data expressed as ng/mg dry extract cannot be directly translated into bitterness thresholds without formulation-specific information. Finally, the fermentation conditions were selected to support the growth of each microorganism; therefore, the results should be interpreted as practical functional fermentation profiles rather than temperature-independent rankings. From a scale-up and sustainability perspective, strain selection may influence not only the functional profile of the fermented product but also process feasibility, including fermentation duration, downstream extraction needs, and the value recovered from lemon processing residues. A strain selected for antioxidant enhancement, flavonoid preservation, or limonoid-associated debittering may therefore lead to different processing routes and economic outcomes within a circular by-product utilization strategy [5,6]. For industrial application, future work should further evaluate product-specific sensory acceptance, especially for *R. stolonifer*-fermented samples with reduced limonin, and compare candidate strains in model beverage, extract, or ingredient formulations to determine whether the observed chemical changes translate into practical sensory and market-relevant benefits [16,48]. Future studies should include enzyme activity assays, untargeted metabolomics, sugar and organic acid profiling, sensory evaluation, product-specific validation, and preliminary techno-economic or life-cycle assessment to confirm the proposed application directions.

## 4. Conclusions

This study demonstrated that fermentation of lemon processing by-products by different food-relevant microorganisms produced distinct remodeling profiles rather than a single uniform biotransformation outcome. Under the common substrate-based framework, *L. plantarum* showed the most consistent antioxidant enhancement profile, with increased hesperetin and phenolic acid responses together with low DPPH and ABTS IC_50_ values. *L. pentosus* promoted flavonoid remodeling but showed a more timing-sensitive antioxidant response. *S. cerevisiae* tended to preserve glycosylated flavonoids and showed a release-oriented phenolic acid profile with strong early ABTS activity. In contrast, *R. stolonifer* exhibited the most pronounced limonoid remodeling, including limonin reduction and obacunone accumulation, suggesting potential relevance for bitterness-oriented applications.

These findings support an application-oriented strain selection framework for lemon by-product valorization: *L. plantarum* may be selected for antioxidant-oriented fermentation, *L. pentosus* for flavonoid conversion with controlled fermentation time, *S. cerevisiae* for preservation-/release-oriented profiles, and *R. stolonifer* for limonoid-associated debittering strategies. However, the proposed mechanisms remain literature-supported interpretations because enzyme activities, untargeted metabolomics, sugar and organic acid profiles, and sensory evaluation were not performed. Future studies should validate these strain-specific profiles through enzyme assays, broader metabolite profiling, product-specific sensory testing, and scale-up assessment.

## Figures and Tables

**Table 1:** Pre-culture media and incubation conditions used for inoculum preparation of the eight microorganisms.

Organism	BCRC Number	Growth Conditions	Oxygen Requirement	Medium
*L. paracasei*	17484	30 °C, 2 days	Facultatively anaerobic	MRS
*L. plantarum*	10357	30 °C, 2 days	Facultatively anaerobic	MRS
*L. pentosus*	12944	30 °C, 1 day	Facultatively anaerobic	MRS
*L. bulgaricus*	10696	37 °C, 3 days	Facultatively anaerobic	MRS
*L. mesenteroides*	12580	30 °C, 2 days	Facultatively anaerobic	MRS
*B. subtilis*	11602	30 °C, 2 days	Aerobic	NB
*S. cerevisiae*	20579	24 °C, 3 days	Aerobic	YPD
*R. stolonifer*	31135	24 °C, 5 days	Aerobic	PDB

Strain numbers follow BCRC (Bioresource Collection and Research Center, Taiwan). MRS = De Man–Rogosa–Sharpe; NB = nutrient broth; YPD = yeast extract–peptone–dextrose medium; PDB = potato dextrose broth. All culture media were purchased from Sigma-Aldrich (St. Louis, MO, USA).

**Table 2:** Viable cell counts of microorganisms cultured in their respective routine media at different incubation times. Values are shown as mean values from three independent cultures. CFU = colony-forming units. *L. paracasei* = *Lactobacillus paracasei*; *L. plantarum* = *Lactobacillus plantarum*; *L. mesenteroides* = *Leuconostoc mesenteroides*; *L. pentosus* = *Lactobacillus pentosus*; *L. bulgaricus* = *Lactobacillus bulgaricus*; *B. subtilis* = *Bacillus subtilis*; *S. cerevisiae* = *Saccharomyces cerevisiae*; *R. stolonifer* = *Rhizopus stolonifer*.

hr/CFU	0	24	48	72	96
*L. paracasei*	3.10 × 10^5^	1.54 × 10^9^	1.22 × 10^9^	5.30 × 10^8^	1.67 × 10^5^
*L. plantarum*	3.2 × 10^5^	2.3 × 10^9^	5.30 × 10^8^	2.08 × 10^8^	2.44 × 10^8^
*L. mesenteroides*	7.00 × 10^5^	7.10 × 10^8^	4.90 × 10^8^	5.60 × 10^7^	1.82 × 10^7^
*L. pentosus*	8.20 × 10^5^	1.10 × 10^9^	1.50 × 10^8^	1.30 × 10^7^	8.00 × 10^5^
*L. bulgaricus*	6.30 × 10^4^	1.10 × 10^9^	6.60 × 10^8^	1.00 × 10^7^	7.00 × 10^6^
*B. subtilis*	1.30 × 10^4^	3.60 × 10^7^	1.50 × 10^7^	2.90 × 10^7^	9.80 × 10^6^
*S. cerevisiae*	3.30 × 10^4^	6.90 × 10^6^	4.20 × 10^7^	6.10 × 10^9^	3.60 × 10^7^
*R. stolonifer*	-	-	-	-	-

**Table 3:** DPPH and ABTS radical-scavenging activities (IC_50_) of extracts from fermented lemon by-products during 7 days of fermentation. IC_50_ values are expressed as mg dry ethanol extract/mL required to achieve 50% radical-scavenging activity. Values are presented as mean ± standard deviation (SD) (*n* = 3). Different lowercase letters indicate significant differences among fermentation days within the same strain (*p* < 0.05). Asterisks indicate significant differences compared with the corresponding unfermented control on the same day (* *p* < 0.05, ** *p* < 0.01, *** *p* < 0.001). Lower IC_50_ values indicate stronger radical-scavenging activity. DPPH = 2,2-diphenyl-1-picrylhydrazyl; ABTS = 2,2′-azino-bis(3-ethylbenzothiazoline-6-sulfonic acid); IC_50_ = half-maximal inhibitory concentration; SD = standard deviation; UFLP = unfermented lemon by-product control; L.pa = *Lactobacillus paracasei*; L.pl = *Lactobacillus plantarum*; L.m = *Leuconostoc mesenteroides*; L.pe = *Lactobacillus pentosus*; L.b = *Lactobacillus bulgaricus*; B.s = *Bacillus subtilis*; S.c = *Saccharomyces cerevisiae*; R.s = *Rhizopus stolonifer*.

Sample		DPPH IC_50_ (mg/mL)	ABTS IC_50_ (mg/mL)
Strain	Day		
UFLP	1	1.34 ± 0.08 ^c^	25.00 ± 1.63 ^ab^
	2	1.81 ± 0.14 ^bc^	21.47 ± 1.21 ^b^
	3	1.80 ± 0.12 ^bc^	23.69 ± 1.21 ^ab^
	4	1.87 ± 0.18 ^b^	24.17 ± 2.25 ^ab^
	5	2.00 ± 0.17 ^b^	25.79 ± 1.75 ^ab^
	6	2.10 ± 0.19 ^b^	26.42 ± 1.51 ^a^
	7	3.60 ± 0.31 ^a^	22.01 ± 1.52 ^ab^
L.pa	1	1.97 ± 0.20 ^c,^**	16.52 ± 1.42 ^d,^**
	2	3.70 ± 0.27 ^a,^***	31.03 ± 2.67 ^ab,^**
	3	3.04 ± 0.25 ^bc,^***	29.94 ± 1.54 ^ab,^**
	4	2.22 ± 0.15 ^c^	27.22 ± 1.38 ^bc^
	5	3.01 ± 0.24 ^b,^**	32.80 ± 2.02 ^a,^**
	6	2.46 ± 0.21 ^bc^	17.11 ± 1.15 ^d,^***
	7	2.85 ± 0.15 ^b,^*	23.78 ± 2.22 ^c^
L.pl	1	2.19 ± 0.14 ^a,^***	16.76 ± 1.28 ^ab,^**
	2	1.68 ± 0.11 ^bd^	15.77 ± 0.91 ^ab,^**
	3	1.70 ± 0.10 ^bc^	15.69 ± 1.21 ^ab,^***
	4	1.42 ± 0.11 ^cde,^*	15.81 ± 0.87 ^ab,^**
	5	1.30 ± 0.07 ^e,^**	14.26 ± 1.12 ^b,^***
	6	1.44 ± 0.13 ^cde,^**	17.91 ± 1.19 ^a,^**
	7	1.78 ± 0.11 ^b,^***	15.86 ± 1.46 ^ab,^**
L.m	1	2.42 ± 0.23 ^a,^**	24.75 ± 2.47 ^a^
	2	1.89 ± 0.10 ^bc^	23.03 ± 1.27 ^ab^
	3	2.33 ± 0.22 ^ab,^*	20.16 ± 1.95 ^bc^
	4	2.81 ± 0.21 ^a,^**	25.27 ± 1.37 ^a^
	5	2.67 ± 0.17 ^a,^**	20.10 ± 1.47 ^bc,^*
	6	1.42 ± 0.12 ^c,^**	16.38 ± 1.19 ^c,^***
	7	1.86 ± 0.16 ^bc,^***	15.86 ± 1.28 ^c,^**
L.pe	1	2.24 ± 0.17 ^c,^***	13.85 ± 1.36 ^d,^***
	2	4.24 ± 0.28 ^ab,^***	27.96 ± 1.82 ^a,^**
	3	4.31 ± 0.42 ^ab,^***	29.56 ± 2.11 ^a,^*
	4	4.40 ± 0.35 ^a,^***	30.01 ± 2.13 ^a,^*
	5	4.31 ± 0.30 ^ab,^***	25.10 ± 1.87 ^ab^
	6	3.99 ± 0.24 ^ab,^***	19.06 ± 1.83 ^c,^**
	7	3.51 ± 0.30 ^b^	21.15 ± 1.21 ^bc^
L.b	1	3.50 ± 0.34 ^a,^***	25.37 ± 1.92 ^b^
	2	3.53 ± 0.32 ^ab,^***	25.56 ± 2.26 ^b^
	3	3.68 ± 0.36 ^ab,^***	28.74 ± 2.41 ^b,^*
	4	3.69 ± 0.33 ^ab,^***	29.89 ± 2.03 ^ab,^*
	5	3.59 ± 0.25 ^ab,^***	28.48 ± 2.58 ^b^
	6	4.23 ± 0.37 ^ab,^***	35.96 ± 3.11 ^a,^**
	7	3.20 ± 0.24 ^b^	25.98 ± 2.12 ^b^
B.s	1	2.19 ± 0.17 ^b,^***	22.98 ± 2.21 ^a^
	2	2.81 ± 0.24 ^a,^**	22.19 ± 1.18 ^a^
	3	2.80 ± 0.18 ^a,^***	20.83 ± 2.02 ^ab^
	4	2.85 ± 0.17 ^a,^**	21.52 ± 1.64 ^ab^
	5	3.21 ± 0.19 ^a,^***	21.03 ± 1.53 ^ab,^*
	6	3.20 ± 0.21 ^a,^**	18.62 ± 1.22 ^ab,^**
	7	2.96 ± 0.22 ^a,^*	17.57 ± 1.23 ^b,^*
S.c	1	2.00 ± 0.19 ^b,^**	11.96 ± 0.69 ^c,^***
	2	2.77 ± 0.25 ^a,^**	14.41 ± 0.74 ^bc,^***
	3	2.76 ± 0.19 ^a,^**	14.61 ± 1.31 ^bc,^***
	4	3.12 ± 0.20 ^a,^***	19.07 ± 1.31 ^a,^*
	5	2.98 ± 0.18 ^a,^**	19.71 ± 1.56 ^a,^*
	6	2.75 ± 0.24 ^a,^*	17.46 ± 1.41 ^ab,^**
	7	2.49 ± 0.20 ^b,^**	13.40 ± 0.92 ^c,^***
R.s	1	1.53 ± 0.15 ^c^	20.16 ± 1.24 ^b,^*
	2	1.56 ± 0.16 ^c^	19.64 ± 1.39 ^b^
	3	3.22 ± 0.21 ^ab,^***	40.83 ± 2.42 ^a,^***
	4	3.15 ± 0.30 ^ab,^**	42.22 ± 2.84 ^a,^***
	5	3.30 ± 0.18 ^a,^***	38.31 ± 3.23 ^a,^**
	6	3.57 ± 0.28 ^a,^**	41.63 ± 4.05 ^a,^**
	7	2.62 ± 0.18 ^b,^**	39.95 ± 3.95 ^a,^**

**Table 4:** Functional classification of strain-specific remodeling profiles during lemon by-product fermentation.

Strain	Dominant Remodeling Profile	Main Supporting Observations	Antioxidant Outcome	Suggested Application Direction
*L. paracasei*	Depletion-oriented profile	Lower limonin, nomilin, and early *p*-coumaric acid decrease	Variable	Limited or secondary candidate
*L. plantarum*	Antioxidant-enhancement type	Strong hesperetin accumulation and active phenolic acid remodeling	Most stable low DPPH and ABTS IC_50_	Antioxidant-oriented fermented extract
*L. mesenteroides*	Weak-to-moderate remodeling type	Minor to moderate changes across metabolite classes	Late-stage improvement	Possible secondary candidate
*L. pentosus*	Flavonoid-conversion, timing-sensitive type	Strong flavonoid conversion and *p*-coumaric acid depletion	Weaker mid-stage antioxidant activity	Flavonoid conversion with controlled fermentation time
*L. bulgaricus*	Limited favorable remodeling type	Limited favorable metabolite changes	Generally weaker antioxidant activity	Less suitable under tested conditions
*B. subtilis*	Moderate remodeling type	Moderate limonoid and flavonoid responses	Moderate antioxidant response	General but non-specific remodeling
*S. cerevisiae*	Preservation/release-oriented type	Hesperidin preservation and phenolic acid release/accumulation	Strong early ABTS response	Preservation of glycosylated flavonoids or early soluble antioxidant response
*R. stolonifer*	Limonoid-remodeling type	Strong limonin reduction and obacunone increase	Late-stage antioxidant loss	Potential debittering-oriented candidate

This table summarizes the dominant strain-specific trends observed across the 7-day fermentation period. It is intended as a functional quick-reference summary for strain selection. For DPPH and ABTS IC_50_, lower values indicate stronger radical-scavenging activity. Suggested application directions are based on targeted metabolite profiles and chemical antioxidant assays and should be validated in future product-specific tests.

## Data Availability

The data presented in this study are available from the corresponding author upon reasonable request.

## Supplementary Materials

The following supporting information can be downloaded at: https://www.mdpi.com/article/10.3390/antiox15060730/s1, Table S1: Detailed day-by-day variation trends of targeted metabolites and antioxidant indices during lemon by-product fermentation by different microbial strains.
