# Supplementary material for: Valorization of Lemon Processing By-Products Through Multi-Strain Fermentation: Strain-Specific Changes in Flavonoids, Limonoids, and Antioxidant Capacity
Source: Antioxidants (Basel). 2026 Jun 9;15(6):730. doi: 10.3390/antiox15060730 (PMC13295453; doi:10.3390/antiox15060730)
Supplement: Supplementary file 1 [file antioxidants-15-00730-s001.zip › antioxidants-4330817-supplementary.pdf]

Supplementary data

Supplementary Table S1. Detailed day-by-day variation trends of targeted metabolites and antioxidant indices during lemon by-product fermentation by different microbial strains.

		Flavonoids			Limonoids			Phenolic acids			Antioxidant	
Compound		Hesperidin	Hesperetin	Narirutin	Nomilin	Obacunone	Limonin	*p*-coumaric acid	Ferulic acid	Vanillic acid	DPPH IC^{50}	ABTS IC^{50}
Strain	Day											
	1	⇊		↑	↑	↑				⇈	↑	↓
	2	⇊	⇈	⇈	↑		↓	↓	⇊	⇈	⇈	↑
	3	⇊	⇈		↑		↓	⇊	↓	↑	⇈	↑
L.pa	4	⇊	⇈	↑	⇊	⇈	↑	↓	⇊	↑		
	5	⇊	↓		↑			↓	↓	↑	↑	↑
	6	⇊	⇈			⇈	↑		↓	⇈		⇊
	7	⇊	⇈		↓	↑	↑	↓	↓	⇈	↓	
	1	⇊	⇈	⇊	↓	⇈	↑		↑	⇈	⇈	↓
	2	⇊	⇈	⇊	↓	⇈	↑	⇊		⇈		↓
	3	⇊	⇈	⇊		⇈	⇈	⇊		⇈		⇊
L.pl	4	⇊	⇈	⇊		⇈	⇈	⇊	↓		↓	↓
	5	⇊	⇈	⇊		⇈	⇈	⇊	⇊	↓	↓	⇊
	6	⇊	⇈	⇊	↓	⇈	⇈	⇊	↓	⇊	↓	↓
	7	⇊	⇈	⇊	↑	↑	⇈	⇊	↑		⇊	↓
	1	↓	↑			↑				⇈	↑	
	2		⇈		↑			↓		⇈		
	3	↓	⇈	⇊		↓		↓		⇈	↑	
L.m	4	↓	⇈		⇊	↑	↑	↓	↓	⇈	↑	
	5				⇊		⇈		⇊	⇈	↑	↓
	6	↓	⇈		↓	⇈	⇈		↓	⇈	↓	⇊
	7		⇈			⇈	⇈		↑	⇈	⇊	↓
	1	⇊	⇈	⇊	⇊	⇈		↓	⇊	⇈	⇈	⇊
L.pe	2	⇊	⇈	⇊	⇊	↑	⇈	⇊	⇊	⇈	⇈	↑
	3	⇊	⇈	⇊	⇊	↑	⇈	⇊	⇊		⇈	↑

	**4**	⇊	⇈	⇊	⇊	⇈	↑	⇊	⇊	⇊	⇈	↑
	**5**	⇊	⇈	⇊	⇊	⇈	⇈	⇊	⇊		⇈	
	**6**	⇊	⇈	⇊		⇈	⇈	⇊	↓		⇈	↓
	**7**	⇊	⇈	⇊	↑	⇈	⇈	⇊	⇊			
	**1**	↓			↓		↓	↓			⇈	
	**2**	↓		⇊	↓		↓	↓			⇈	
	**3**	↓		⇊	↑		⇊	↓	↑	↓	⇈	↑
L.b	**4**	↓	↓	↓		⇊	↑	↓		⇊	⇈	↑
	**5**		⇊			⇊	⇈	↓	↓		⇈	
	**6**	↑		↓	↑		⇈				⇈	↑
	**7**	↓	↓	↓	⇈	⇊	↑	↓	↑			
	**1**	⇊		↓	⇊	⇈		↓			⇈	
	**2**	↓	↓	↓	↓	⇈	↑	⇊		↑	↑	
	**3**	⇊		⇊	↑	⇈	↑	⇊	↑		⇈	
B.s	**4**	↓	↑	↓	↓	⇈	⇈		↓	↑	↑	
	**5**				⇊	⇈	⇈		⇊	⇈	⇈	↓
	**6**		↑	↓	↓	⇈	⇈		↓		↑	↓
	**7**	↓	⇈	↓	↑	⇈	⇈	⇊		↑	↓	↓
	**1**	⇊	↑	⇊	⇊	⇈			↑	↑	↑	⇊
	**2**	↑	⇈		↑	↑	↑		↑	↑	↑	⇊
	**3**	↓	⇈	⇊	⇈	⇈	⇈		⇈	↓	↑	⇊
S.c	**4**			↓	↑	↑	⇈		↑		⇈	↓
	**5**		↑		↑	⇈	⇈	⇈			↑	↓
	**6**		⇈		⇈	⇈	⇈	↑	↑		↑	↓
	**7**		⇈	↓	⇈	↑	⇈	↓	⇈		↓	⇊
	**1**	⇊	⇈	⇊		⇈		⇊	⇈	⇈		↓
	**2**	⇊	⇈	⇊	↑			⇊	⇈	↑		
	**3**	⇊	⇈	⇊	↑	↑	⇈	⇊	↑	⇊	⇈	⇈
R.s	**4**	⇊	⇈	⇊	↓	↑	↑	⇊	⇈	⇊	↑	⇈
	**5**	⇊	⇈	⇊	⇊	↑	⇈	⇊	⇈	⇊	⇈	↑
	**6**	⇊	⇈	⇊	↓	⇈	↑	⇊	⇈	⇊	↑	↑
	**7**	⇊	⇈	⇊	↓	⇈	↑	⇊	⇈	⇊	↓	↑

This table summarizes the direction and statistical significance of changes in targeted flavonoids, limonoids, phenolic acids, and antioxidant indices during the 7-day fermentation period. Each fermented sample was compared with the corresponding unfermented lemon by-product control (UFLP) at the same sampling day. Arrows indicate the direction and level of change: ↑ denotes a significant increase ($p < 0.05$), ↓ denotes a significant decrease ($p < 0.05$), whereas ⇈ and ⇊ represent highly significant increases and decreases ($p < 0.001$), respectively. Blank cells indicate no significant change based on the applied

statistical criteria. For antioxidant indices (DPPH and ABTS IC50), the arrow direction refers to the IC50 value; therefore, ↓ indicates enhanced radical-scavenging capacity, whereas ↑ indicates reduced radical-scavenging capacity.
